# Efficacy and Safety of Paragastric Neural Blockade in Controlling Pain, Nausea, and Vomiting After Sleeve Gastrectomy: A Randomized Controlled Trial

**DOI:** 10.1007/s11695-024-07255-9

**Published:** 2024-05-07

**Authors:** Mehmet Kağan Katar, Umut Fırat Turan

**Affiliations:** General Surgery Department, Faculty of Medicine, Atlas University, Istanbul, 34450 Turkey

**Keywords:** Sleeve gastrectomy, Paragastric neural blockade, Pain block, Postoperative pain, Postoperative nausea and vomiting, Visceral block

## Abstract

**Background:**

There are difficulties in controlling the symptoms of pain, nausea, and vomiting after laparoscopic sleeve gastrectomy (LSG). This study aimed to evaluate the efficacy and safety of PGNB on pain and nausea and vomiting in the early postoperative period in patients who underwent LSG.

**Methods:**

In this prospective, randomized, controlled, double-blind study, the patients were divided into two equally formed groups: patients who underwent PGNB after LSG and the control group. Postoperative pain symptoms were evaluated using the visual analog scale (VAS) scores, and nausea and vomiting symptoms were evaluated using the postoperative nausea and vomiting (PONV) scores.

**Results:**

The study was completed with 90 patients, 45 patients in each group. The VAS scores measured at postoperative hours 1, 6, and 12 were statistically significantly lower in the PGNB group. There was no significant difference between the two groups in terms of the 24th hour VAS scores. The mean PONV scores of the PGNB and control groups were 0.47 ± 0.89 and 1.67 ± 1.95, respectively, revealing a significantly higher value for the controls. The mean time to first mobilization in the postoperative period was significantly shorter in the PGNB group. Upon the evaluation of patient satisfaction, it was determined that the satisfaction score of the PGNB group was significantly higher.

**Conclusions:**

PGNB is an effective and safe method for managing pain, nausea, and vomiting that occur in the early period after LSG.

**Graphical Abstract:**

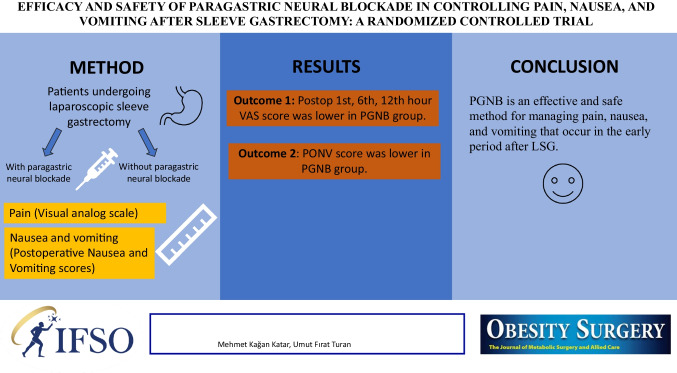

## Introduction

Obesity and its associated disorders have significant implications for health, resulting in heightened productivity loss and reduced life expectancy while also negatively impacting the quality of life of patients [1]. Surgical intervention is the most effective way to achieve sustainable weight loss in patients with obesity and to alleviate the associated comorbidities.

The annual incidence of surgical treatments for obesity management is steadily rising, with approximately 580,000 individuals undergoing obesity surgery across the world each year [2]. Despite the current availability of minimally invasive bariatric surgical treatments, postoperative pain remains a significant issue. Postoperative pain is divided into two groups: somatic pain and visceral pain [3]. Various techniques are employed to manage postoperative somatic pain, including transversus abdominis plane (TAP) block, port site injection, and erector spinae plane (ESP) block [4–7]. While these methods are effective in controlling somatic pain, they have no effect on visceral pain. On the other hand, opioid-derived drugs, which are mostly used to control somatic pain, can lead to respiratory depression and constipation [8, 9] and adversely affect the quality of life of patients in the early postoperative period. In order for patients to have a healthier postoperative period, enhanced recovery after surgery (ERAS) protocols recommend reducing opioid use after bariatric surgery [10].

Many methods, such as celiac plexus, splanchnic nerve, superior and inferior hypogastric, and ganglion impar blocks, have been evaluated to control abdominal and pelvic pain in patients with chronic visceral pain of benign or malignant origin [11]. The celiac plexus provides sympathetic, parasympathetic, and visceral sensory afferent fibers to the upper abdominal viscera, including the pancreas, liver, bile ducts, gallbladder, spleen, adrenal glands, kidneys, mesentery, stomach, small intestine, and the proximal part of the transverse colon [12]. Research has demonstrated that patients’ pain and opioid consumption decrease in diseases such as chronic pancreatitis and pancreatic cancer, especially after the celiac plexus block procedure [13].

Vagal and sympathetic afferent signals originating from the gastrointestinal system stimulate the vomiting center, causing nausea and vomiting. Following laparoscopic sleeve gastrectomy (LSG), nausea and vomiting may occur as a result of increased intraluminal pressure in the stomach due to the decreased extensibility and compliance of the postoperative gastric pouch [14]. Therefore, any intervention that reduces intraluminal pressure can prevent nausea and vomiting.

Paragastric neural blockade (PGNB) is a new method performed by injecting local anesthetic material into three to four separate points along the border between the lesser omentum and the stomach (from the esophagogastric junction to the distal antrum), the hepatoduodenal ligament, and the area covering the left gastric artery. This procedure aims to prevent visceral pain and symptoms of nausea and vomiting [15]. This study aimed to evaluate the efficacy and safety of PGNB on pain and nausea and vomiting in the early postoperative period in patients who underwent LSG.

## Methods

### Study Design, Setting, and Participants

This prospective, randomized, controlled, double-blind study was carried out between May 2023 and July 2023. The study was initiated after receiving approval from the ethics committee of Atlas University (IRB E-22686390–050.99–27,043) and conducted in accordance with the tenets of the Declaration of Helsinki. Following the approval of the ethics committee, the study was registered at ClinicalTrials.gov (ID: NCT05984160).

The sample consisted of individuals aged 18–65 years with a body mass index (BMI) over 35 kg/m^2^ and an obesity-related comorbidity or those with a BMI over 40 kg/m^2^, who were scheduled to undergo LSG, had an American Society of Anesthesiologists (ASA) risk score of 2–3 and agreed to participate in the study.

Excluded from the study were patients with chronic pain disorders, those using gabapentin, opioid addicts, patients using anticoagulant drugs, those with a history of previous upper gastrointestinal system surgery, those with surgery-related complications during or after surgery, those with liver or kidney failure, those with moderate or severe cardiovascular or respiratory problems, those who were allergic to the local anesthesia agent to be administered during the PGNB procedure, those with limited cooperation, those who required more than one surgical intervention in the same session, and those who were allergic to the drugs to be used in postoperative management.

An informed volunteer consent form was signed by all patients who agreed to participate in the study. No additional tests were performed on the patients other than routine preoperative biochemical tests and whole abdominal ultrasonography.

### Outcomes and Variables

The State-Trait Anxiety Inventory for Adults was used to evaluate the preoperative anxiety levels of the patients included in the study [16]. In addition, the visual analog scale (VAS) (0–10 points) was administered to the patients at hours 1, 6, 12, and 24 to evaluate their postoperative pain severity, and the postoperative nausea and vomiting (PONV) questionnaire was administered at hours 6 and 24 to evaluate their nausea and vomiting symptoms [17]. A pain assessment was undertaken, while the patients were in bed, after resting for at least 10 min. The heart rates and blood pressures of the patients were recorded before PGNB and 10 min after the procedure. Whether the patients required additional analgesics or antiemetic drugs and the time to first mobilization were also noted. Lastly, patient satisfaction with surgical experience was evaluated based on a Likert-type scale (0–5 points) before their discharge from the hospital.

### Anesthesia Protocol

Anesthesia was induced with 2.5–3.5 mg/kg of propofol, 1 μg/kg of fentanyl, and 0.6 mg/kg of rocuronium bromide and maintained with 2% sevoflurane and a remifentanil infusion in 100% oxygen. The remifentanil infusion was started at a dose of 0.1 μg/kg/min and titrated up to 0.4 μg/kg/min as required during surgery. Thirty minutes before the end of surgery, 4 mg of ondansetron, 8 mg of dexamethasone, and 50 mg of dexketoprofen trometamol were intravenously administered to all patients. During awakening, inhalation anesthesia was turned off, and the muscle relaxant effect was reversed by administering 0.05 mg/kg of neostigmine and 0.01 mg/kg of atropine. The patients were then extubated and transferred to the recovery room.

### Surgical Technique and PGNB Procedure

All operations were performed by two surgeons experienced in the field of obesity surgery (M.K.K., U.F.T.). Pneumoperitoneum was established using a 12-mm optical trocar. Using a camera advanced through this site, a 12-mm working port was placed on the patient’s right, as well as three 5-mm trocars (a liver retractor, a working port from the left side, and an assistant port). The greater curvature of the stomach was freed using the LigaSure™ (Maryland–Medtronic, Minneapolis, MN) energy device. A resection of the stomach was performed, starting at 4 cm from the pylorus, using a 38-French bougie. The leak test was performed with methylene blue diluted with physiological saline. Then, the PGNB procedure was applied. Reinforcement stitches were placed on the stapler line with 3/0 V-Loc™ sutures (Covidien, Mansfield, MA, USA).

PGNB was performed intraoperatively once the resection of the stomach was completed. The blockade was applied with a short 25-gauge needle attached to a venous catheter extension inserted through a 12-mm port. The needle cap was on during insertion and removed from the abdomen using a holder, being kept under constant monitoring. A total of 18 mL of undiluted 0.5% bupivacaine was applied to the fatty tissues in the posterosuperior paragastric area covering the left gastric artery by exposing the esophagogastric junction, proximal stomach, mid-stomach, distal antrum, hepatoduodenal ligament, and posterior stomach along the border of the lesser omentum, ensuring that 3 cc of the agent reached each of these six regions. Intravenous injection was avoided by aspiration before injection. The cap was then reattached to the needle, and the assembly was removed from the abdominal cavity [15].

### Postoperative Follow-Up and Treatment

Postoperatively, patients were routinely administered intravenous (IV) hydration, 40 mg of IV pantoprazole (every 24 h), 10 mg of IV metoclopramide HCl (every 8 h), 1,000 mg of IV paracetamol (every 6 h), 50 mg of IV dexketoprofen trometamol (every 8 h), and enoxaparin sodium (every 24 h) according to their weight. For patients with a VAS score of > 4, if it was not routine treatment time, 100 mg of IV tramadol hydrochloride was administered as the first-line rescue analgesic, and 100 mg of IV pethidine hydrochloride was administered as the second-line rescue analgesic. For those whose complaints of nausea and/or vomiting continued despite routine treatment, 8 mg of IV ondansetron was administered as an additional antiemetic. The day following the surgery, oral intake of clear food was initiated in all patients.

### Sample Size Calculation

A power analysis was performed to determine the number of patients to be included in the study. Using the descriptive statistics reported in the article entitled “Paragastric Autonomic Neural Blockade to Prevent Early Visceral Pain and Associated Symptoms After Laparoscopic Sleeve Gastrectomy: a Randomized Clinical Trial,” the effect size was calculated to be 0.744, and the minimum number of patients required was determined to be 78 (39 in each group) to achieve a 95% confidence level (a = 0.05) and 90% power (Hintze, J.(2011). PASS 11. NCSS, LLC. Kaysville, Utah, USA. www.ncss.com.) Considering possible losses, a total of 90 patients were included in the study.

### Randomization

The patients were divided into one of two equally formed groups: group 1 included patients who underwent the PGNB procedure, and group 2 included controls. A randomization scheme was created on the website www.randomization.com. Randomization was performed by a general surgeon who was not present during the PGNB procedure. The patients were numbered sequentially according to the randomization scheme. These numbers were recorded in follow-up files. Postoperative results were evaluated by a general surgeon who was blinded to the patient groups. Since both the patients and the general surgeon who would perform the evaluation did not know whether the PGNB procedure had been performed, the trial was conducted in a double-blind manner.

### Statistical Analysis

While analyzing the findings obtained from the study, the Statistical Package for the Social Sciences (SPSS) for Mac, v. 26.0 was used for statistical analysis. Descriptive statistics were presented as mean and standard deviation for quantitative variables and numbers and percentages for qualitative variables. Analytical methods (Kolmogorov–Smirnov/Shapiro–Wilk test) were used to determine whether continuous variables were normally distributed. The Mann–Whitney *U* test was used for non-normally distributed numerical data (non-parametric method), while Student’s *t*-test (parametric method) was used for normally distributed numerical data. Relationships between categorical variables were analyzed using the chi-square test. *p* < 0.05 was considered statistically significant.

## Results

**Fig. 1:**
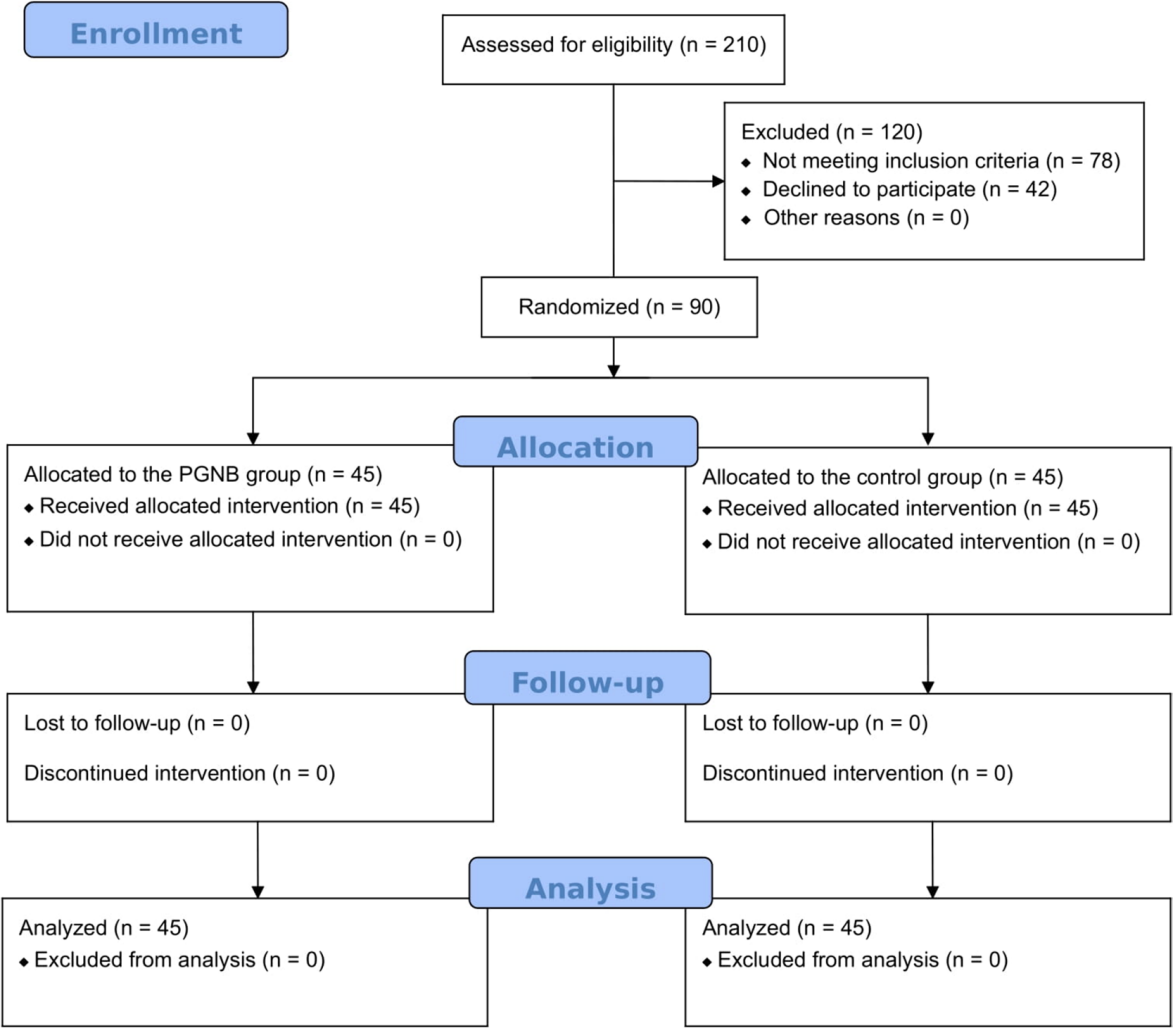
Flow chart of study

The study included a total of 90 patients who met the inclusion criteria. The PGNB and control groups consisted of 45 patients each. No patient was lost to follow-up during the study (Fig. 1). In our analysis, we found no statistically significant difference between the two groups in terms of age, gender, BMI, comorbidities, or a history of abdominal surgery (Table 1).

**Table 1:** Patient demographics and baseline characteristics

	PGNB group (*n* = 45)	Control group (*n* = 45)	*p* value
Age (year)	38.93 ± 10.03	35.78 ± 7.45	.094
Gender			
Female	32 (71.1)	37 (82.2)	.213
Male	13 (28.9)	8 (17.8)	
BMI	44.05 ± 5.96	42.68 ± 6.39	.147
Anxiety score	76.36 ± 17.66	78.00 ± 16.90	.653
Comorbidities			
Diabetes	10 (22.2)	6 (13.3)	.270
Hypertension	16 (35.6)	17 (37.8)	.827
Hyperlipidemia	18 (40)	16 (35.6)	.664
Obstructive sleep apnea	3 (6.7)	4 (8.9)	.694
History of abdominal surgery	14 (31.1)	20 (44.4)	.192

Values are presented as mean ± standard deviation or number (%). Anxiety score: The State-Trait Anxiety Inventory for Adults score

*BMI* body mass index, *PGNB* paragastric neural blockade

### Postoperative Pain

The comparison of the two groups in terms of pain intensity revealed that the VAS scores measured at postoperative hours 1, 6, and 12 were statistically significantly lower in the PGNB group than in the control group. Although the mean VAS score at postoperative hour 24 was lower in the PGNB group than in the control group, this did not create a statistical difference. Detailed data concerning the VAS scores are given in Table 2.

**Table 2:** Comparison of postoperative VAS scores

	PGNB group	Control group	*p* value
VAS-hour 1	3.09 ± 1.52	5.44 ± 2.31	< .001
VAS-hour 6	3.07 ± 2.00	4.89 ± 2.15	< .001
VAS-hour 12	3.00 ± 1.93	4.38 ± 2.07	.002
VAS-hour 24	2.96 ± 1.79	3.64 ± 1.68	.060

Values are presented as mean ± standard deviation

*VAS* visual analog scale, *PGNB* paragastric neural blockade

First-line rescue analgesics were administered to eight (17.8%) patients in the PGNB group and 33 (73.3%) patients in the control group, indicating a statistically significantly higher rate of first-line rescue analgesic requirement in the latter (*p* < 0.001). Similarly, the need for second-line rescue analgesics was found to be significantly higher in the control group compared to the PGNB group [two (4.4%) patients in the PGNB group and 14 (31.1%) patients in the control group, *p* = 0.001] (Table 3).

**Table 3:** Postoperative outcomes

	PGNB group	Control group	*p* value
First-line rescue analgesic requirement	8 (17.8)	33 (73.3)	< .001
Additional antiemetic requirement	5 (11.1)	16 (35.6)	.006
Operation time	58.04 ± 7.46	55.20 ± 9.77	.075
Time to first mobilization	174.33 ± 57.15	201.22 ± 57.88	.018
PONV score	0.47 ± 0.89	1.67 ± 1.95	.001
Satisfaction score	4.22 ± 0.87	3.53 ± 0.75	< .001

Values are presented as mean ± standard deviation or number (%)

*PGNB* paragastric neural blockade, *PONV* postoperative nausea and vomiting

### Postoperative Nausea and Vomiting

The mean PONV scores of the PGNB and control groups were determined to be 0.47 ± 0.89 and 1.67 ± 1.95, indicating a significantly higher score for the controls (*p* = 0.001). It was also found that five (11.1%) patients in the PGNB group and 16 (35.6%) patients in the control group required additional antiemetics in the postoperative period, with this requirement being at a significantly higher rate in the control group (*p* = 0.006) (Table 3).

### Operation Time and Efficacy of PGNB

Although the mean operation time of the PGNB group (58.04 ± 7.46 min) was higher than that of the control group (55.20 ± 9.77 min), there was no statistically significant difference (*p* = 0.075) (Table 3). In the PGNB group, the mean pulse, systolic blood pressure (SBP), and diastolic blood pressure (DBP) values were determined to be 78.98 ± 13.27 beats/min, 115.29 ± 15.66 mmHg, and 65.27 ± 10.72 mmHg, respectively, before the procedure and 73.42 ± 11.56 beats/min, 104.69 ± 12.986 mmHg, and 60.71 ± 9.49 mmHg, respectively, 10 min after the procedure. According to this evaluation, the values of these three parameters determined 10 min after the procedure were significantly lower compared to those obtained before the procedure (*p* < 0.001 for all).

### Complications

Four patients who underwent the PGNB procedure developed a localized hematoma in the injection area where the blockade was applied. In all these patients, the hematoma was controlled with local compression, and no additional intervention was required. Except for hematoma, no other complication related to the PGNB procedure developed in any of the patients.

### Postoperative Mobilization Time and Patient Satisfaction

Our analysis showed that the mean time to first mobilization in the postoperative period was significantly shorter in the PGNB group than in the control group (*p* = 0.018). Concerning the evaluation of patient satisfaction, the satisfaction score of the PGNB group was significantly higher compared to the control group (*p* < 0.001) (Table 3).

## Discussion

Various methods are employed to control postoperative pain following LSG, including the application of TAP and ESP blocks and the administration of local anesthetics to the incision site [5, 18]. However, these approaches exclusively target somatic nerves, thereby effectively managing only somatic pain that occurs due to trauma to the anterior abdominal wall during surgery [19] while being insufficient to control visceral pain and alleviate other visceral symptoms [20]. According to the results of our study, PGNB significantly reduced visceral pain that occurred within the first 24 h after LSG. This was achieved without a significant prolongation of operation time or the development of any major complications. Injection-related local hematoma occurred in only four patients and was resolved after a few minutes of compression in all cases. In contrast, serious complications have been reported after block procedures, especially following the administration of TAP and ESP blocks. As an example, pneumothorax and intravascular injection have been reported after ESP block, while liver injury has been described after TAP block [21–23]. Future comparative studies can reveal whether PGNB is superior to other methods in terms of efficacy in both eradicating postoperative pain and eliminating the risk of complications.

The data obtained from our study revealed that, due to less postoperative pain in patients undergoing PGNB, their opioid requirement in the postoperative period was lower compared to the control group. By decreasing the use of opioids, we may be able to prevent certain potential opioid-related complications, including increased risk of respiratory depression, sedation, airway obstruction, and prolonged hospital stay [24–26].

Nausea and vomiting, which occur especially within the first 24 h after LSG, still pose a serious problem despite all the pharmacological therapies used [27, 28]. In fact, according to a study conducted by Suh et al., nausea and vomiting that occurred after LSG caused prolonged hospital stays and an increased number of emergency department visits after discharge from the hospital [29]. While some studies recommend using prophylactic pharmacological agents to prevent postoperative nausea and vomiting, others recommend other agents for treatment [30–33]. Nevertheless, it is evident that there is not yet a consensus in the literature concerning the solution to the problem in question. On the other hand, in our study, PGNB significantly reduced the complaints of nausea and vomiting that occurred within the first 24 h after LSG. Consequently, PGNB also reduced the need for additional antiemetics. A decrease in the requirement for additional antiemetics also means a reduced risk of complications related to the use of these agents.

Our findings showed that the time to first postoperative mobilization was shorter in patients who underwent PGNB compared to those who did not undergo this procedure. One possible explanation for this finding is the use of opioid medications, which are commonly prescribed to alleviate postoperative pain within the initial hours after surgery in individuals who did not undergo PGNB. Opioids are generally used as analgesic drugs in the early postoperative period, and one of the most common side effects of this group of drugs is sedation [34, 35]. Therefore, we consider that opioids used to relieve pain may have delayed the patients’ first mobilization due to their sedative effects in the early postoperative period. Another possible reason is that patients who underwent PGNB had fewer pain complaints in the early postoperative period. Specifically, less pain in these patients may have reduced their movement restrictions, encouraging them to actively participate in first mobilization. Furthermore, mobilization of patients early can help prevent various possible complications in the cardiovascular system, gastrointestinal system, and musculoskeletal system associated with immobilization [36].

Our findings indicate a significant reduction in the pulse, SBP, and DBP values of the PGNB group 10 min after the procedure, as compared to the pre-procedure measurements. We attributed this decrease to sympathetic inhibition. Therefore, these parameters can be used as markers to evaluate whether PGNB is performed effectively.

In this study, we also found that patients who underwent PGNB had higher levels of satisfaction with their surgical experience. This is probably due to these patients having less pain and experiencing less nausea and vomiting in the early postoperative period.

We consider that our study provides a new perspective on addressing the challenges in the management of pain, nausea, and vomiting after LSG, which remain subjects of ongoing discussion. In addition, since this is the first randomized, controlled study on this subject, it will serve as a basis for future research.

### Limitations

A limitation of this study is that the PGNB procedure is contingent upon the skill and experience of the surgeon. While it may appear to be a simple procedure, it is crucial to ensure that the injection is performed accurately and adequately.

## Conclusion

PGNB is an effective and safe method for managing pain, nausea, and vomiting that occur in the early period after LSG. In addition to reducing the need for opioids and antiemetics in the early postoperative period, it also facilitates the earlier mobilization of patients.
